# Unlocking the potential of molecular-driven stratification for osteosarcoma treatment and prognosis

**DOI:** 10.18632/oncotarget.28364

**Published:** 2023-02-11

**Authors:** Gaël Moquin-Beaudry, Maria Eugenia Marques da Costa, Nathalie Gaspar, Antonin Marchais

**Keywords:** multiomics, osteosarcoma, bone sarcoma, stratification

Over the last 40 years, the complex genetic landscape, the heterogeneity of the microenvironment and the cell plasticity of Osteosarcoma (OSA) tumors have delayed the therapeutic and prognostic stratification of patients and the introduction of new efficient treatments. As a direct consequence, the vast majority of trials still don’t benefit from a selection of OSA patient based on molecular evidence before drug administration. This lack of stratification leads to difficult interpretation of outcome, especially with targeted agents such as multikinase inhibitors or anti-osteoclastic drugs.

Meanwhile, fortunately, the accumulation of numerous sparse but converging observations from many research and clinical teams have progressively drawn a portrait of the resistant osteosarcoma that’s paved the way to new translational discoveries.

Recently, several important studies have described OSA molecularly at an unprecedent level of detail taking advantage of multiomics approaches and artificial intelligence [1–3]. In a recent study, our team used unsupervised machine learning algorithms to classify OSA at diagnosis based on gene expression modules functionally enriched for immune microenvironment and tumor phenotypic traits [1]. Strikingly, the unsupervised two-group classification was strongly and significantly associated to survival. Our classification recapitulated sparse observations about the biology of OSA: osteoclastogenesis, angiogenesis, presence of neutrophils and cancer associated fibroblasts as poor prognosis markers, and pro-inflammatory immune infiltrate and the expression of specific cancer testis genes as favorable prognosis markers. These results allow us, already at diagnosis, to identify patients which are least likely to respond to current treatments, thus likely to benefit from new therapeutic combinations. Like other similar promising studies however, our work still suffers from the “small cohort size” curse of rare diseases and a difficulty to identify the exact biological determinant or proxy related to the gene expression signature inferred by machine learning, especially from bulk Omics dataset. Powerful state of the art approaches like single cell [4, 5] and spatial transcriptomics will allow for more thorough dissection of disease mechanisms and study of relevant intercellular interactions with a diagnostic or therapeutic translational potential, but are even more prone to cohort under sampling. The next challenge in OSA will be to increase the statistical power of such studies by combining datasets throughout the world. The building of such data collections requires multilevel structure frameworks with national, regional and international initiatives. For instance, our group supervises the French BoOSTDataS and the European FOSTER consortia and participates to international HIBISCus consortium and the global pediatric Human Cell Atlas initiative to ensure that researchers have access to the best quality and quantity of data which are essential for the field to move forward and improve treatments for patients. We are very enthusiastic about these initiatives and the recent rate of discoveries in OSA, and hopeful that they will bring actionable targets for OSA.
